# A Rare Case of 15q11.2 Microdeletion Syndrome with Atypical Features: Diagnostic Dilemma

**DOI:** 10.7759/cureus.3543

**Published:** 2018-11-05

**Authors:** Waliul Chowdhury, Pooja Patak, Farjahan J Chowdhury, Hasnan M Ijaz, Tehmina Zafar, Nick Chatla, Ahmad Khiami

**Affiliations:** 1 Internal Medicine, Raleigh General Hospital, Beckley, USA; 2 Pediatrics, Raleigh General Hospital, Beckley, USA

**Keywords:** microdeletion syndrome, 15q11.2 microdeletion syndrome, chromosomal microarray analysis

## Abstract

Burnside Butler syndrome or 15q11.2 microdeletion syndrome is a relatively rare chromosomal abnormality that is recently being recognized. Current diagnostic techniques like chromosomal microarray analysis (CMA) have profoundly contributed to currently reported cases. The diagnostic dilemma is that prenatal screening and karyotype analysis typically yield unclear results. We would like to emphasize the importance of taking a detailed family history, knowing the classic clinical features, and using CMA to help diagnose this syndrome. We present an eight-year-old Caucasian female with a past medical history of intrauterine growth restriction, microcephaly, a high arched palate, speech delay, and a learning disability with recurrent bleeding from the eyes and oral cavity. The bleeding co-occurs whenever she develops the common cold.

## Introduction

The typical features of Burnside Butler syndrome include neurobehavioral problems, developmental and language delays, intrauterine growth restriction, and dysmorphic features [1-2]. Prenatal screening and karyotype analysis are typically not helpful for this diagnosis [1]. However, a karyotype analysis should still be used initially to rule out common chromosomal abnormalities, such as Down syndrome [3]. Hence, we would like to place emphasis on the diagnostic methods used for our patient and her family, and present her atypical features. We would also like to present the consequences of having an additional microdeletion (1p21.3), which was absent in the patient but present in her mother and maternal half-brother, both of whom had the 15q11.2 microdeletion.

## Case presentation

History

An eight-year-old Caucasian female presented with intermittent bleeding from the eyes and oral cavity, which started when she was two years old. Her bleeding would be exacerbated by symptoms of the common cold. Based on similar bleeding that occurred in both her sister and maternal half-brother, the family expected that the bleeding would stop at one year of age. In kindergarten, she was diagnosed with a learning disability and was reported to be 17 months behind in her developmental goals. She was transferred to special education classes for further assistance.

Her birth history included a birth weight of four pounds (lb) and four ounces (oz) (1927.77 grams) and a premature gestational age of 36 weeks. Her head circumference at birth was below the fifth percentile, consistent with microcephaly. She was in the neonatal intensive care unit (NICU) for four weeks due to intrauterine growth restriction and severe respiratory distress. A cesarean section was done due to maternal bleeding and a previous cesarean section. Her mother reported her first words were at around three years of age. According to her past medical records, she presented with grunting and tachypnea at birth. She continued to grunt despite 30% oxygen therapy, which subsided after three hours. Her group B streptococcus testing was negative. She had feeding issues during the first six days after birth and lost six ounces of weight. A nasogastric feeding tube was used for an additional three days. There was an innocent heart murmur detected at birth, which shortly subsided. This similarly occurred in both her maternal half-brother and sister at birth.

The patient had one sister and one maternal half-brother. Her sister had nearly the same appearance as the patient, as described in the physical exam. Her brother and mother had autistic-like features. Her maternal half-brother’s neurocognitive function progressively worsened after three years of age. Her mother had a severe intellectual disability, attention deficit hyperactivity disorder (ADHD), and myopia. The patient’s sister also had microcephaly, which was not present in her maternal half-brother or mother. There was also a maternal uncle with mild mental retardation. Her father also had mild mental retardation, a learning disability, and ADHD. There was no other history of congenital disabilities, mental retardation, or any known genetic disease on either side of the family. The parents were Caucasian and non-consanguineous.

Physical exam

The current vital signs were as follows: temperature 97.1 degrees Fahrenheit (ºF), pulse 84 beats per minute, respiratory rate 21 breaths per minute, and blood pressure 100/68 millimeters of mercury (mmHg). Her body mass index was 13; height was four and a half feet, and weight was 44.8 pounds. Her current audiometry testing at age eight showed a score of 500/4000 in both ears. Her appearance was prominent for a narrow-shaped face with thin, brittle hair, short stature, an extremely thin habitus, deep-set eyes, and diffuse muscular atrophy. The rest of her physical exam was unremarkable.

Diagnostic testing

**Figure 1:**
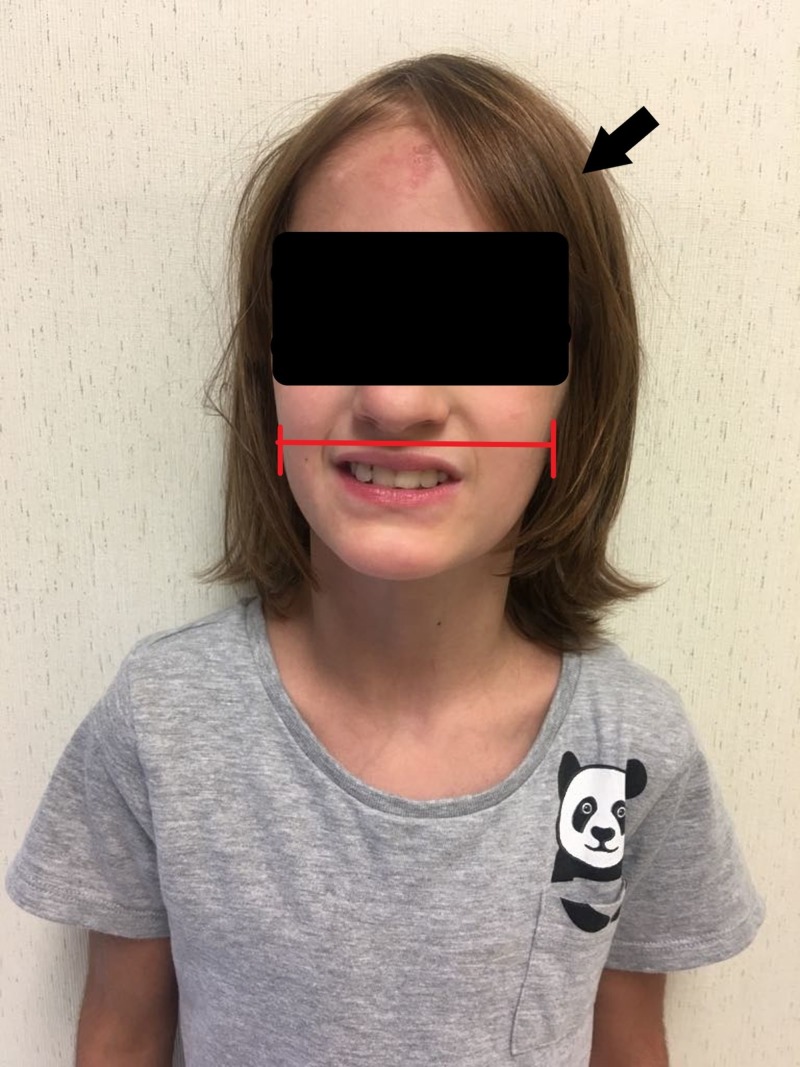
Frontal view of the patient demonstrating fine hair (black arrow) and a narrow face (red bracket)

**Figure 2:**
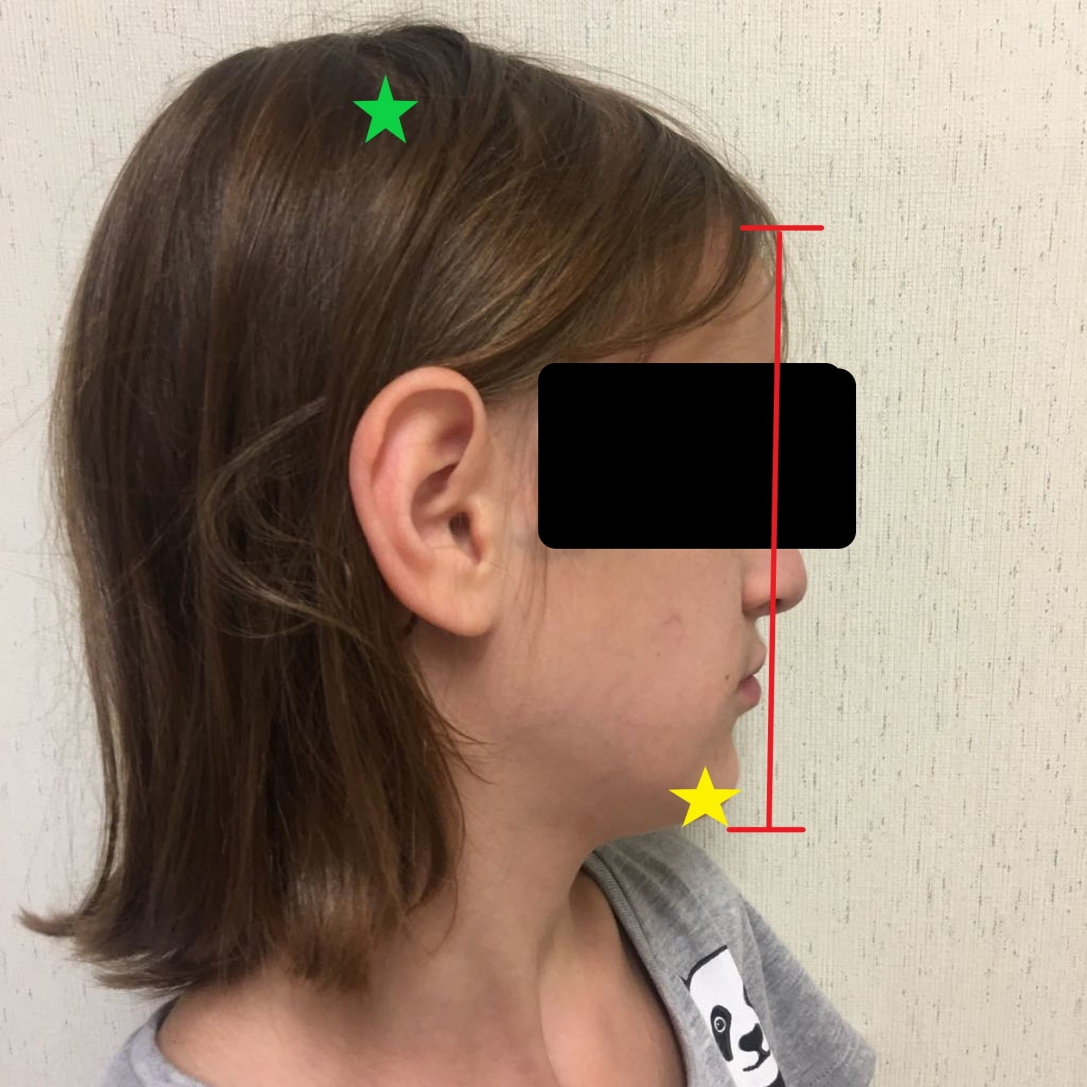
Lateral view of the patient showing a narrow-shaped skull (green star), micrognathia (yellow star), and a flat facial profile (red bracket)

**Figure 3:**
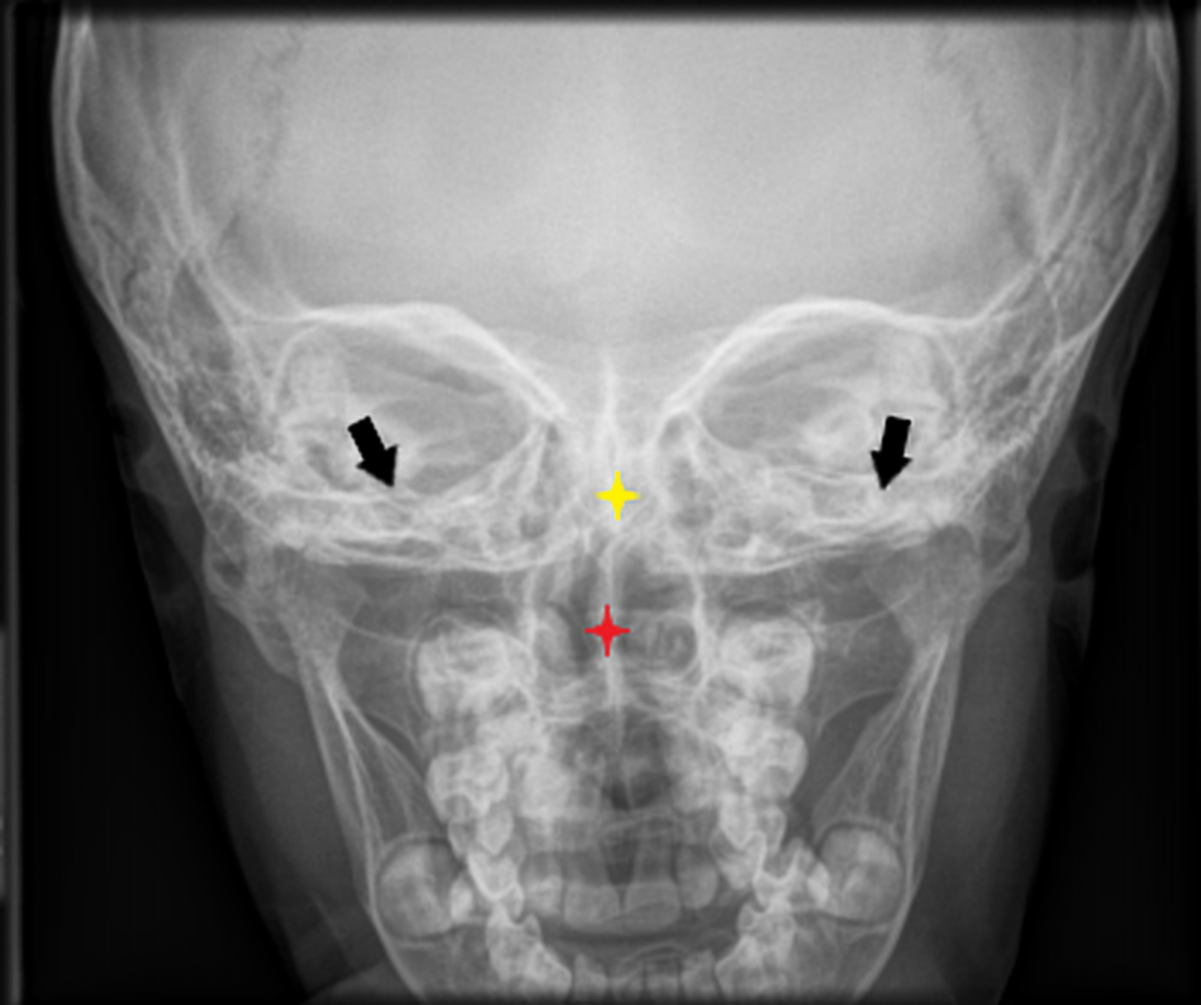
Frontal X-ray imaging of the face at age 5, which shows a slightly deviated nasal septum to the right (red star) but no structural abnormalities or fractures Yellow star = cribriform plate, red star = nasal septum, black arrows = inferior orbital plate

**Figure 4:**
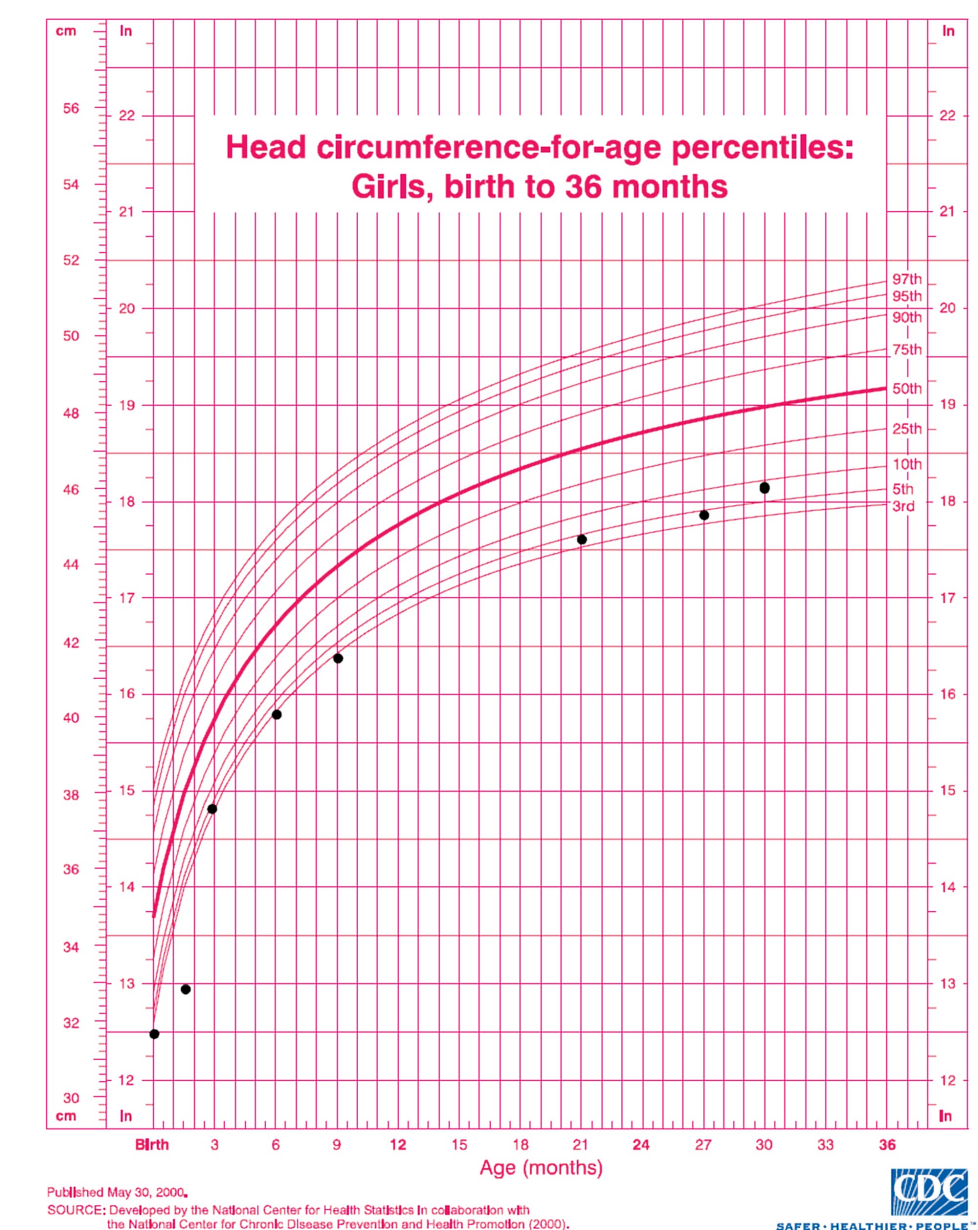
Head circumference trend (black circles) from birth to 36 months. The trend shows an abnormally low head circumference relative to age, which started trending upward starting at 27 months of age

**Figure 5:**
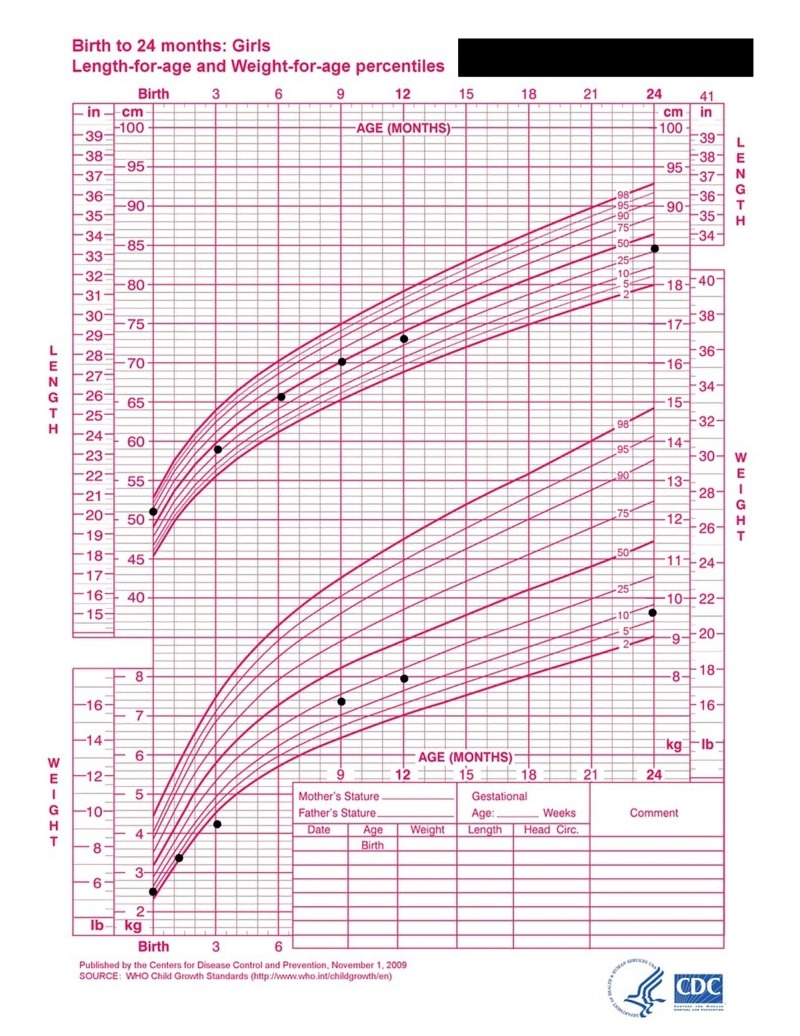
Weight and length trend (black circles) from birth to 36 months. The results show an abnormally low body weight relative to length

**Figure 6:**
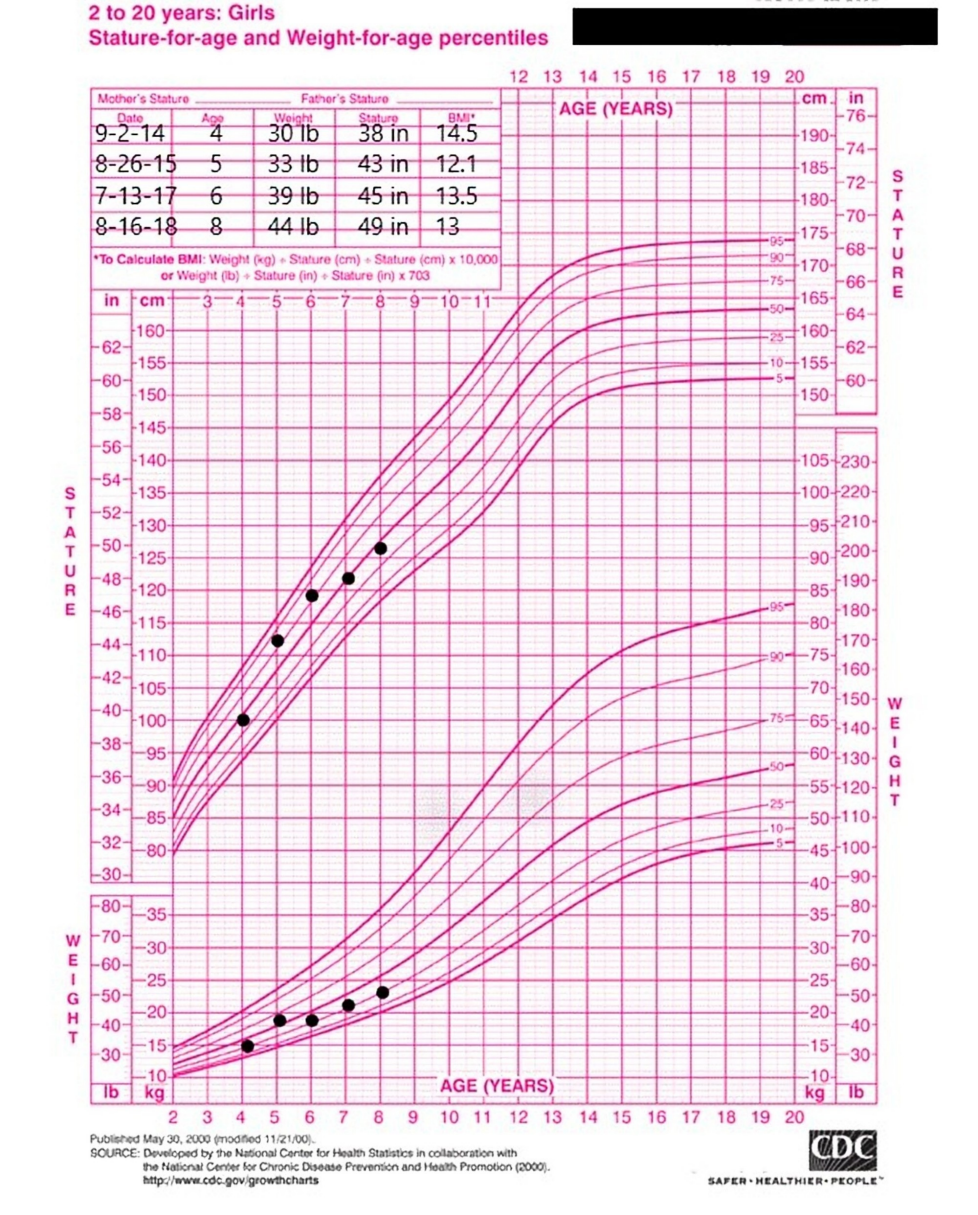
Weight and stature trend from two years to current age. The trend shows an abnormally lower body weight relative to stature and a consistently low body mass index (BMI)

Images of the patient's anterior and lateral face are shown in Figures 1-2, respectively. Figure 1 demonstrates the patient's fine hair and narrow-shaped face. Figure 2 shows the patient's flat facial profile, narrow-shaped skull, and micrognathia. These features added to the suspicion of a possible chromosomal abnormality. An X-ray image of the patient’s anterior face ruled out morphological abnormalities or fractures leading to the patient's recurrent bleeding, as shown in Figure 3. The patient's head circumference and weight and length trends from birth to 24 months and from 24 months to eight years are shown in Figures 4-6, respectively. The patient's neonatal screen results were normal, as shown in Table 1. Coagulation testing and complete blood count results were also normal, as shown in Tables 2-3, respectively. Platelet function testing showed low levels of adenosine diphosphate (ADP) expression. She was referred for genetic testing at four months of age. All of her immediate family members were also evaluated. A comparative genomic hybridization (CGH) microarray analysis was done and showed that the patient, her mother, sister, and maternal brother all had the 15q11.2 microdeletion. The 15q interval was flanked by segmental repeats; breakpoint segments one and two (BP I and II). The region included four, non-imprinted, highly conserved genes: TUBGCP5, NIPA1, NIPA2, and CYFIP1. The breakpoint start position was 20, 301, and 966. The breakpoint end position was 20, 779, and 211. The minimum size of the deleted segment was 477 Kb. Her mother and maternal brother also had a second chromosome deletion at chromosome 1p21.3. The patient and her sister did not have this second chromosome deletion. No other significant deoxyribonucleic acid (DNA) copy number changes or copy neutral loss of heterozygosity (LOH) was detected in the 1,800,000 region-specific single nucleotide polymorphisms (SNP). Fluorescent in situ hybridization (FISH) assay was also done on the patient and family members. However, the results were inconclusive. An electroencephalogram (EEG) was also conducted on the patient and showed no seizure activity.

**Table 1:** Initial neonatal screening collected five days after birth showing normal results. µg/dL= microgram per deciliter, µIU/mL = micro international units per milliliter, mg/dL = milligrams per deciliter

Disorder	Result	Remarks	Expected values
Congenital hypothyroidism (CH)	Normal	Within normal limits	T4 >6.8 µg/dL and <30 µg/dL TSH <20 µIU/mL
Hemoglobinopathy	Fetal hemoglobin and adult hemoglobin (FA)	Normal newborn	FA
Galactosemia	Normal	Within normal limits	Total galactose (TGAL) <10 mg/dL
Biotinidase deficiency	Normal	Within normal limits	Normal activity
Congenital adrenal hyperplasia	Normal	Within normal limits	Within normal limits
Cystic fibrosis (CF)	Normal	Within normal limits	Lower 95%
Amino acid profile	Normal	Within normal limits	Within normal limits
Fatty acid profile	Normal	Within normal limits	Within normal limits
Organic acid profile	Normal	Within normal limits	Within normal limits

**Table 2:** Coagulation testing at age eight showing normal results s = seconds

Test	Result	Reference
Prothrombin (PT) time	11.1 s	9.6-11.8 s
International normalized ratio (INR)	1.04	0.89-1.12
Partial thromboplastin time (PTT)	25 s	25-35 s

**Table 3:** Complete blood count at age eight showing normal results K/mm^3^ = thousand per millimeter cubed, mL/mm = milliliter per millimeter, g/dL = grams per deciliter, UM3 = Units meter cubed, pg = picograms

Test	Result	Reference
White blood cell (WBC)	9.2	6.2-19.9 K/mm^3^
Red blood cell (RBC)	4.16	3.70-4.90 mL/mm
Hemoglobin (Hb)	11.7	10.7-13.5 g/dL
Hematocrit (Hct)	35.5	31.6-39.5%
Mean corpuscular volume (MCV)	85	77.3-88.3 UM^3^
Mean corpuscular hemoglobin (MCH)	28	26.3-30.3 pg
Mean corpuscular hemoglobin concentration (MCHC)	33	33.2-34.8%
Red cell distribution width (RDW)	12.2	11.5-14.5%
Platelet count	360	140-450 K/mm^3^

## Discussion

Chromosome 15q11.2 microdeletion syndrome is becoming more recognized, with a prevalence of 0.57%-1.27% [1]. The microdeletions occur between chromosomal breakpoints one and two (BP1-BP2) along the long arm (q) of chromosome 15; position 11.2. This critical region contains four non-imprinted genes: NIPA1, NIPA2, CYFIP1, and TUBGCP5. These genes are located in the central nervous system (CNS). Consequently, these patients typically present with psychomotor and speech delays, developmental delays, dysmorphic features, autism, attention deficit hyperactivity disorder (ADHD), and seizures [1-2]. The inheritance pattern is autosomal dominant, with a variation in phenotype and incomplete penetrance [1].

Knowing the clinical features is key in raising clinical suspicion and diagnosing 15q11.2 microdeletion syndrome. A literature review by Cox et al. included 200 patients diagnosed with 15q11.2 microdeletion syndrome using CMA [1]. They analyzed the clinical features based on their compiled data. These features included growth and developmental abnormalities, dysmorphic features, intelligence quotient (IQ), academic achievement, behavioral and psychiatric issues. The general developmental delay was by far the most common feature, seen in 73% of the patients. Speech and motor delay was seen in 67% and 42% of patients, respectively. Microcephaly was seen in 24% of patients. General dysmorphic features were present in 39% of patients and abnormalities of the palate were seen in 46% of the individuals. Neurocognitive abnormalities were writing difficulties (60%), memory problems (60%), reading difficulties (50%), and a verbal IQ score of 75 or less (50%). Other associations were ADHD (35%), autism spectrum disorder (27%), oppositional defiant disorder (24%), obsessive-compulsive disorder (26%), and schizophrenia or paranoid psychosis (20%). Aside from these symptoms, 43% of individuals had abnormal brain imaging, with features of epilepsy (26%) [1]. Recognizing these clinical features in a child coupled with a detailed history are crucial for diagnosing this syndrome. If clinical suspicion is raised, diagnostic screening tests like CMA should subsequently be done.

The chromosome microarray analysis is a relatively new diagnostic technique and is highly accurate in diagnosing many rare chromosomal abnormalities. The International Standard Cytogenomic Array Consortium conducted a literature review of 33 studies, which included 21,698 patients, and compared the use of a CMA with a G-band karyotype analysis in terms of diagnostic yield, technical advantages, and disadvantages. Results showed that CMA was 15%-20% more accurate in diagnosing unexplained autism spectrum disorder, developmental disabilities, intellectual disabilities, and multiple congenital anomalies. This excluded patients with Down syndrome and other well-known chromosomal syndromes. They also showed that CMA could not accurately diagnose truly balanced gene rearrangements and syndromes containing low-level mosaicism. The authors strongly suggested using CMA over G-type karyotyping for unexplained developmental or intellectual disabilities, autism spectrum disorders, or multiple congenital anomalies. However, a G-banded karyotype analysis should be used over CMA for patients with apparent chromosomal syndromes like Down syndrome, patients with a family history of known chromosomal rearrangements, or a history of multiple miscarriages [3].

An interesting feature of the patient was her recurrent bleeding from the eyes and mouth exacerbated by periods of the common cold. Her sister and maternal half-brother with the same 15q11.2 microdeletion syndrome also presented with similar symptoms. The patient and her siblings all had normal coagulation test results, which makes this an unusual presentation. To our knowledge, there is no reported association of 15q11.2 microdeletion syndrome with inherited bleeding disorders in the current literature. Therefore, we cannot state that bleeding is an associated feature of 15q11.2 microdeletion syndrome. Future case reports with unusual bleeding as part of their presentation may provide stronger evidence of a relationship.

Congenital heart defects are a rare, yet essential, association of 15q11.2 microdeletion syndrome. For example, a familial case of total anomalous pulmonary venous return associated with 15q11.2 was recently reported. A comparative genomic hybridization (CGH) microarray analysis results showed a 395 kilobyte (kb) deletion at 15q11.2, confirmed with fluorescence in situ hybridization in two of the three siblings and their asymptomatic father [4]. In addition, a case study of 52 patients with 15q11.2 microdeletion syndrome showed that 17.3% of the patients had congenital heart defects [5]. Recognizing these associations and providing early genetic counseling may reduce future cardiac risk.

Comparative genomic hybridization works by breaking down DNA into two sample sizes of experimental and control groups. Both samples are dyed with different colors to distinguish which part of the chromosome is deleted or duplicated. In our case, the region located at 11.2 of the long arm (q) of chromosome 15 was deleted, recognized by the reduced resolution of the specific metaphase chromosomes compared with the control sample [6].

As described in the case presentation, neither the patient nor her sister had an additional deletion at chromosome 1p21.3, but her mother and maternal step-brother did. Coincidentally, the patient's mother and maternal step-brother presented with more severe intellectual disability in terms of speech and learning impairment, which progressively worsened with age. Her mother and maternal step-brother also had severe autism spectrum disorder (ASD), which was not present in the patient or her sister. The additional microdeletion on chromosome 1p21.3 may have contributed to the severe intellectual disability in the mother and maternal step-brother. A study by Carter et al. identified four individuals from three unrelated families with hemizygous deletions on chromosome 1p21.3 involving the dihydropyrimidine dehydrogenase (DPYD) gene [7]. DPYD is the rate-limiting enzyme involved in the breakdown of pyrimidine bases [7-8]. All four patients were autistic with severe speech delay [7]. Further studies are needed to state an association between 15q11.2 and 1p21.3 microdeletion syndromes and its effect on cognitive dysfunction.

## Conclusions

Burnside Butler (15q11.2 microdeletion) syndrome is a rare, autosomal, dominant chromosomal abnormality with a broad range of clinical features, which makes it difficult to diagnose. Clinicians should recognize the key features associated with this syndrome such as developmental, speech, and motor delay, cognitive dysfunction, and dysmorphic features like a high, arched palate, narrow face and skull, fine hair, micrognathia, and sunken eyes. Recognizing these abnormal features along with a detailed family history should raise clinical suspicion, and clinicians should subsequently undergo a CGH microarray analysis. However, a karyotype analysis should still be done initially to exclude common chromosomal abnormalities. An association to keep in mind is congenital heart defects. There may be additional chromosomal deletions in family members with this syndrome, which may contribute to cognitive variation among the family. Further studies are needed to outline the underlying pathophysiology of this syndrome, and detailed diagnostic guidelines are needed. With the use of chromosomal microarray analyses, more data can hopefully be compiled in the future to explain this syndrome in more detail.
